# Mechanical CPR devices compared to manual CPR during out-of-hospital cardiac arrest and ambulance transport: a systematic review

**DOI:** 10.1186/1757-7241-20-39

**Published:** 2012-06-18

**Authors:** Marcus Eng Hock Ong, Kevin E Mackey, Zhong Cheng Zhang, Hideharu Tanaka, Matthew Huei-Ming Ma, Robert Swor, Sang Do Shin

**Affiliations:** 1 Department of Emergency Medicine, Singapore General Hospital, Outram Road, Singapore, 169608, Singapore; 2 Department of Emergency Medicine, Kaiser Permanente, Sacramento, CA, USA; 3 Department of Emergency System, Graduate School of Sport System, Kokushikan University, Tokyo, Japan; 4 Department of Emergency Medicine, National Taiwan University, Taipei, Taiwan; 5 Department of Emergency Medicine, William Beaumont Hospital, Royal Oak, MI, USA; 6 Department of Emergency Medicine, Seoul National University Hospital, Seoul, Korea

**Keywords:** “Mechanical”, “Automatic”, “Load distribution band, “Cardiopulmonary resuscitation”, “Chest compression”, “Transport”, and “Transportation”

## Abstract

**Aims:**

The aim of this paper was to conduct a systematic review of the published literature to address the question: “In pre-hospital adult cardiac arrest (asystole, pulseless electrical activity, pulseless Ventricular Tachycardia and Ventricular Fibrillation), does the use of mechanical Cardio-Pulmonary Resuscitation (CPR) devices compared to manual CPR during Out-of-Hospital Cardiac Arrest and ambulance transport, improve outcomes (e.g. Quality of CPR, Return Of Spontaneous Circulation, Survival)”.

**Methods:**

Databases including PubMed, Cochrane Library (including Cochrane database for systematic reviews and Cochrane Central Register of Controlled Trials), Embase, and AHA EndNote Master Library were systematically searched. Further references were gathered from cross-references from articles and reviews as well as forward search using SCOPUS and Google scholar. The inclusion criteria for this review included manikin and human studies of adult cardiac arrest and anti-arrhythmic agents, peer-review. Excluded were review articles, case series and case reports.

**Results:**

Out of 88 articles identified, only 10 studies met the inclusion criteria for further review. Of these 10 articles, 1 was Level of Evidence (LOE) 1, 4 LOE 2, 3 LOE 3, 0 LOE 4, 2 LOE 5. 4 studies evaluated the quality of CPR in terms of compression adequacy while the remaining six studies evaluated on clinical outcomes in terms of return of spontaneous circulation (ROSC), survival to hospital admission, survival to discharge and Cerebral Performance Categories (CPC). 7 studies were supporting the clinical question, 1 neutral and 2 opposing.

**Conclusion:**

In this review, we found insufficient evidence to support or refute the use of mechanical CPR devices in settings of out-of-hospital cardiac arrest and during ambulance transport. While there is some low quality evidence suggesting that mechanical CPR can improve consistency and reduce interruptions in chest compressions, there is no evidence that mechanical CPR devices improve survival, to the contrary they may worsen neurological outcome.

## Background

Sudden cardiac arrest is a global concern. Over the years, the mechanism of death has shifted from ventricular fibrillation (VF) as initial rhythm to pulseless electrical activity (PEA) and asystole [1-4]. The incidence of out-of-hospital cardiac arrest (OHCA) in USA has been estimated at 1.89/1,000 person-years and at 5.98/1,000 person-years in subjects with any clinically recognized heart disease [5]. Published survival rates for OHCA ranged from 3.0% to 16.3% in North America [6].

CPR during OHCA and ambulance transport is a key issue in pre-hospital emergency care. CPR in a moving ambulance is a particular challenge. Patients may arrest during transport, or OHCA patients may need to be transported due to local ambulance policies. For example, in Asia-Pacific countries, societal and social norms see pronouncement of death in residences and public places as taboo. Thus, cardiac arrest patients are often transported to the hospital in a ‘scoop and run’ model. This is in contrast to the practice in North American Emergency Medical Services (EMS) systems and European communities, where CPR is more often performed at scene, and unsuccessful resuscitations may be terminated in the field.

The quality of CPR during OHCA is a key factor affecting survival [7]. The problem with standard CPR is that it provides only one third of normal blood supply to the brain and 10-20% of normal blood flow to the heart [8]. Good quality CPR is an even greater challenge in the setting of a moving ambulance. It is also increasingly recognized that although defibrillation is the definitive treatment for ventricular fibrillation, its success is also dependent on adequate circulation [9-11]. Thus, effective CPR is often a prerequisite for effective defibrillation.

Mechanical CPR is an attractive alternative, due to theoretical advantages including elimination of the rescuer fatigue factor, more consistent and reliable chest compression, and eliminating the need to stop CPR during rescuer changes and patient transfers. It is also conceivably safer for the ambulance crew during transport. However results from clinical trials have been conflicting, with studies suggesting a benefit for mechanical CPR [12] and others that failed to find any significant difference between manual and mechanical CPR in survival to discharge [13]. Possible explanations for these unexpected results advanced by the authors included a Hawthorne effect, prolonged deployment time for the devices resulting in delayed defibrillation and enrollment bias [13].

The aim of this paper was to conduct a systematic review of the published literature on the use of mechanical CPR devices compared to manual CPR during OHCA and ambulance transport in pre-hospital adult cardiac arrest regardless of presenting rhythm. This review was conducted as part of a consensus meeting on ambulance CPR, organized by the Asian EMS Council in 2011 (Seoul, Korea). More information about the council can be found at: http://www.scri.edu.sg/index.php/paros-clinical-research-network.

## Methods

The review was conducted in accordance with the methodology recommended by the International Liaison Committee on Resuscitation (ILCOR) 2010 evidence evaluation process [14] where this sought to identify evidence to address the question: [15] “In pre-hospital adult cardiac arrest (asystole, pulseless electrical activity, pulseless VT and VF)(pre-hospital [OHCA]), does the use of mechanical CPR devices compared to manual CPR during ambulance transport, improve outcomes (e.g. quality of CPR, return of spontaneous circulation (ROSC), survival)”.

A search strategy was pursued, using the following search terms: “mechanical”, “automatic”, “load distribution band”, “cardiopulmonary resuscitation”, “chest compression”, “transport” and “transportation” (textword and MeSH headings when applicable).

Databases were searched up to 1 October 2011, with PubMed, the Cochrane Library (including Cochrane database for systematic reviews and Cochrane Central Register of Controlled Trials), Embase, and the American Heart Association (AHA) Resuscitation Endnote Master library, which contains over 15,000 cardiac arrest related references. Moreover, cross-references from articles and reviews were forward searched using SCOPUS and Google scholar.

In addition, we looked at the following review articles: a) Manual versus Mechanical Chest compression for cardiac arrest (Cochrane Review) [16], b) CPR techniques and devices: 2010 International Consensus on Cardiopulmonary Resuscitation and Emergency Cardiovascular Care Science with Treatment Recommendation (AHA) [17]. Cross references from these review articles were forward searched using SCOPUS and Google Scholar.

Inclusion criteria were human studies of adult cardiac arrest (age ≥ 16) and manikin studies which were peer-reviewed. Exclusion criteria were case reports and case series. We broadened the scope of our review from CPR solely during ambulance transport to OHCA in general due to the absence of clinical outcome studies addressing this particular setting.

The articles were reviewed for relevance independently by two reviewers (MEHO/KM). Both titles and abstracts were reviewed, followed by the articles if suitable for review. Articles where the content was clearly unrelated were discarded. The abstracts of remaining articles were then reviewed and relevant studies identified for detailed review of the full manuscript. Where disagreement existed between reviewers, articles were included for detailed review. Finally, the reference lists of narrative reviews were examined to identify any additional articles not captured by the main search strategy.

### Evidence appraisal

Studies were reviewed in detail and then classified by level of evidence (LOE) (Table 1) and quality (rated poor, fair or good) according to agreed definitions (Table 2). “Methodological quality” (internal validity) of a study was defined as “the extent to which a study's design, conduct, and analysis has minimized selection, measurement, and confounding biases” [14]. That quality is separate to “non-methodological” quality, which refers to the external validity or generalizability of the study results to other (broader) population groups.

**Table 1:** International Liaison Committee on Resuscitation (ILCOR) 2010 Levels of Evidence for Studies of Therapeutic Interventions

LOE 1:	Randomized Controlled Trials (or meta-analyses of RCTs)
LOE 2:	Studies using concurrent controls without true randomization (e.g. “pseudo”-randomized)
LOE 3:	Studies using retrospective controls
LOE 4:	Studies without a control group (e.g. case series)
LOE 5:	Studies not directly related to the specific patient/population (e.g. Different patient/population, animal models, mechanical models etc.)

**Table 2:** Quality assessment for studies assessing interventions

LOE 1	**Quality assessment for Randomized Controlled Trials**
	The seven factors included as the relevant quality items for RCTs are:
	· Was the assignment of patients to treatment randomized?
	· Was the randomization list concealed?
	· Were all patients who entered the trial accounted for at its conclusion?
	· Were the patients analyzed in the groups to which they were randomized?
	· Were patients and clinicians "blinded" to which treatment was being received?
	· Aside from the experimental treatment, were the groups treated equally?
	· Were the groups similar at the start of the trial?
	**Quality assessment for meta-analyses of RCTs**
	The six factors included as the relevant quality items for meta-analyses are:
	· Were specific objectives of the review stated (based on a specific clinical question in which patient, intervention, comparator, outcome (PICO) were specified)
	· Was study design defined?
	· Were selection criteria stated for studies to be included (based on trial design and methodological quality)?
	· Were inclusive searches undertaken (using appropriately crafted search strategies)?
	· Were characteristics and methodological quality of each trial identified?
	· Were selection criteria applied and a log of excluded studies with reasons for exclusion reported?
LOE 2	**Quality assessment for studies using concurrent controls without true randomization**
	The four factors included as the relevant quality items for these studies are:
	· Were comparison groups clearly defined?
	· Were outcomes measured in the same (preferably blinded), objective way in both groups?
	· Were known confounders identified and appropriately controlled for?
	· Was follow-up of patients sufficiently long and complete?
	**Quality assessment for meta-analyses of studies using concurrent controls without true randomization**
	The six factors included as the relevant quality items for meta-analyses are:
	· Were specific objectives of the review stated (based on a specific clinical question in which patient, intervention, comparator, outcome (PICO) were specified)
	· Was study design defined?
	· Were selection criteria stated for studies to be included (based on trial design and methodological quality)?
	· Were inclusive searches undertaken (using appropriately crafted search strategies)?
	· Were characteristics and methodological quality of each trial identified?
	· Were selection criteria applied and a log of excluded studies with reasons for exclusion reported?
LOE 3	**Quality assessment for studies using retrospective controls:**
	The four factors included as the relevant quality items for these studies are:
	· Were comparison groups clearly defined?
	· Were outcomes measured in the same (preferably blinded), objective way in both groups?
	· Were known confounders identified and appropriately controlled for?
	· Was follow-up of patients sufficiently long and complete?
LOE 4	**Quality assessment for case series**
	The three factors included as the relevant quality items for these studies are:
	· Were outcomes measured in an objective way?
	· Were known confounders identified and appropriately controlled for?
	· Was follow-up of patients sufficiently long and complete?
LOE 5	**Quality assessment for studies that are not directly related to the specific patient/population**
	LOE 5 studies are those not directly related to the specific patient/population (e.g. different patient/population, animal models, mechanical models etc.), and should have their methodological quality allocated to the methodology of the study. The relevant quality criteria here are:
	· Good = randomized controlled trials (equivalent of LOE 1)
	· Fair = studies without randomized controls (equivalent of LOE 2–3)
	· Poor = studies without controls (equivalent of LOE 4).

Studies were allocated a rating for methodological quality (Good, Fair or Poor) according to the presence of the quality items for that Level of Evidence (see Table 2). Good studies had most/all of the relevant quality items, Fair studies had some of the relevant quality items and Poor studies had few of the relevant quality items (but sufficient value to include for further review).

## Results

Out of 88 articles identified, 10 studies met the inclusion criteria for further review. Of these 10 articles, 1 was LOE 1, 4 LOE 2, 3 LOE 3, 0 LOE 4, 2 LOE 5 (see Table 3).

**Table 3:** Summary of evidence

**Level of Evidence**	**1**	**2**	**3**	**4**	**5**
**Evidence SUPPORTING Clinical Question**					
Good					Stapleton [18], Sunde [19]
Fair		Axelsson [20], Dickinson [21]	Olasveengen [22], Ong [12], Casner [23]		
Poor					
**Evidence NEUTRAL Clinical Question**					
Good					
Fair					
Poor		Axelsson [24]			
**Evidence OPPOSING Clinical Question**					
Good	Hallstrom [13]				
Fair		Wang [25]			
Poor					

Table 4 is a summary of our review of all eligible studies.

**Table 4:** Review of eligible studies

**S/N**	**Title**	**Description of Subjects**	**Study Design**	**Outcomes**	**Results**	**Conclusion**
1	Comparing CPR ambulance transport: Manual versus Mechanical methods [18]	144 simulated runs conducted on a manikin.	Prospective observational study comparing between both types of CPR in different environment settings such as vehicle type, road conditions, vehicle speed.	Compression adequacy (defined as 1.5 to 2 inches in depth, 60 compressions per min +/- 10% and correct hand position).	Thumper provided pre-defined correct compressions for 97% of the runs (n = 70) while manual compressions were performed correctly 37% of the runs (n = 27).	The device is able to deliver higher number of effective and consistent compression at correct position as compared to manual CPR.
					97% rate for the device remains valid for different environment settings while 37% rate for manual CPR varied in different settings.	
			Thumper was used.			
2	Quality of mechanical, manual standard and active compression-decompression CPR on the arrest site and during transport in a manikin model [19]	36 runs conducted on a manikin which runs are split between 3 groups.	Randomized, cross-over, out-of-hospital study comparing between three type of CPR at cardiac arrests sites and during transportation.	Compression depth, frequency, compression – decompression ratio based on European Resuscitation Council Guidelines (ERC).	Compression depth, frequency and compression ratio in mechanical CPR device were within recommended guidelines during ambulance transports as compared to other 2 groups.	Mechanical CPR adhered more closely to ERC guidelines. Working conditions for standard CPR in ambulance transport is undesirable.
3	Quality of cardiopulmonary resuscitation before and during transport in out-of hospital cardiac arrest [22]	Adult patients over 18 years of age suffering from non-traumatic OHCA for all causes and received CPR before and during transportation.	Retrospective, observational study.	Chest compression and ventilation rates, hands-off ratio (time without compressions divided by total CPR time).	Hands-off ratio were lower for mechanical CPR both before (p =0.016) and during transport (p =0.002).	For manual CPR, Time without chest compressions during transport and compression rate was lower due to difficult conditions. As such, the device is more appropriate. No conclusion is drawn about patient outcomes due to small sample size.
			LUCAS was used.			
					Compression rates for mechanical CPR were closer to recommended guidelines (100/min) while manual CPR was higher (96/min vs. 119/min).	
		Manual CPR during transport: 66				
		Mechanical CPR during transport: 9			No difference in CPC scores between two groups.	
4	Video-recording and time motion analysis of manual versus mechanical cardiopulmonary resuscitation during ambulance transport [25]	Adult patients over 18 years of age with OHCA for all rhythms.	Prospective and randomized study where video- recording and time- motion studies was conducted.	Chest compression rate, instantaneous compression rates, unnecessary no chest compression interval, based on guidelines in ILCOR.	No-chest compression interval (Mechanical: 33.4% vs. Manual: 31.63%, p =0.160).	Due to small sample size, short ambulance transport (average 4 minutes) and difficulty in setting/removal of device, it is hard to identify potential benefits of mechanical CPR.
		Manual: 12				
		Mechanical: 8				
					Average chest compression rate excluding no chest compression interval (mechanical: 52.3/min vs. manual: 113.3/min, p <0.050).	
			Thumper was used.			
					Lower ventilation rate excluding no chest compression interval (mechanical 11.7/min vs. manual 16.1/min, p < 0.050). Higher variability in chest compression rates for manual CPR.	
5	The impact of a New CPR Assist Device on rate of return of spontaneous Circulation in Out of hospital Cardiac Arrest [23]	Adult patients with OHCA. No exclusions listed.	Retrospective case–control study with matched control subjects.	ROSC upon arrival at Emergency Department (Asytole/ Agonal, PEA and VT/VF).	ROSC for case matched subjects in all rhythms (mechanical: 39% versus manual: 29%, p = 0.003), Asytole/agonal (mechanical: 37% vs. manual: 22%, p = 0.008), PEA (mechanical: 38% vs. manual: 23%, p =0.079) and VF/VT (no difference).	Mechanical CPR may improve ROSC and benefit patients with non-shockable rhythms.
		Manual: 138				
		Mechanical: 124				
			All mechanical CPR subject groups received manual CPR prior to deployment of the device.			
			AutoPulse was used.			
6	Use of an Automated, Load-distributing Band Chest Compression Device for Out-of-Hospital Cardiac Arrest Resuscitation [12]	Adult patients over 18 years of age with non- traumatic OHCA.	Phased, non-randomized observational study of clinical outcomes of patients treated before and after transition from manual CPR to using mechanical CPR device, AutoPulse.	ROSC, survival to hospital admission and hospital discharge, and neurological outcome at discharge.	ROSC (mechanical: 34.5% vs. manual: 20.2%), Survival to hospital admission (mechanical: 20.9% vs. manual: 11.1%), Survival to hospital discharge (mechanical: 9.7% vs. manual: 2.7%). No difference in CPC (p = 0.360) and OPC (p = 0.400) Better mechanical CPR performance if ambulance response time is less than 8 minutes.	Mechanical CPR is better.
		Manual: 499				
		Mechanical: 210				
7	Manual Chest Compression vs. Use of Automated chest compression device during resuscitation following out of hospital cardiac arrest: a randomized trial [13]	Adult patients over 18 years of age, non-traumatic OHCA and resuscitation attempted by EMS.	Multi-centre randomized trial.	Survival with spontaneous circulation 4 hours after emergency call is made, Survival to hospital discharge, CPC at hospital discharge.	No difference in survival to 4 hours (mechanical: 29.5% vs. manual: 28.5%, p =0.740), survival to discharge (mechanical: 5.8% vs. manual: 9.9%, p =0.060). Difference in CPC score at hospital discharge (mechanical: 3.1% vs. manual: 7.5%, p = 0.006).	Study was terminated when it was found that mechanical CPR was associated with worse neurological outcomes and a trend towards worse survival than manual CPR.
			AutoPulse was used.			
		Manual: 517				
		Mechanical: 554				
8	Clinical consequences of the introduction of chest compression in the EMS system for treatment of out-of-hospital cardiac arrest – a pilot study [24]	Adult patients over 18 years of age, witnessed, non-traumatic OHCA.	Non-randomized, controlled trial. LUCAS was used.	ROSC at any time during treatment, Survival at hospital admission, survival to hospital discharge with neurological recovery.	No significant difference in ROSC, survival to hospital admission or hospital discharge.	No sufficient evidence to support that Mechanical CPR would improve outcome. This was due to long delay between arrival of ALS vehicle and use of Mechanical CPR (6 minutes).
		Manual: 169			No difference in CPC scores between 2 groups (majority 1 and 2).	
		Mechanical: 159				
9	Mechanical active compression-decompression cardiopulmonary resuscitation (ACD-CPR) versus manual CPR according to pressure of end tidal carbon dioxide (P_ET_CO_2_) during CPR in out-of-hospital cardiac arrest (OHCA) [20]	Adult patients over 18 years of age, non-traumatic OHCA.	Prospective, cluster level, pseudo-randomized trial.	ETCO_2_ was measured after tracheal intubation for 15 minutes or until detection of ROSC. Readings were categorized to initial, minimum, average and maximum.	ETCO_2_ was significantly higher in mechanical CPR as compared to manual CPR according to initial (3.38 vs. 2.71, p = 0.010), average (3.26 vs.2.69, p = 0.040) and minimum (2.24 vs. 1.69, p = 0.010). No significant differences according to maximum value between 2 groups (4.88 vs.4.48, p = 0.230). No differences in survival outcomes and there was a long time interval between cardiac arrest to start of CPR and to ROSC for both groups.	Mechanical CPR performed compressions with higher cardiac output than manual chest compressions.
			LUCAS was used.			
		Manual: 62				
		Mechanical: 64				
						
10	Effectiveness of mechanical versus manual chest compressions in out- of-hospital cardiac arrest resuscitation: a pilot study [21]	Adult patients over 16 years of age with non-traumatic OHCA.	Prospective, odd/even day randomization study.	ETCO_2_ measurements at 5 minutes after intubation and CPR began, and a second measure at initiation of transport to hospital.	Statistical significant between both groups. 80% in manual group has decreasing ETCO_2_ while 20% has increasing ETCO_2_.	Mechanical CPR produced better cardiac output, overall, ;when compared to manual CPR.
			Odd days were using mechanical CPR and even days using manual CPR.			
		Manual: 10				
		Mechanical:10				
					All patients (3 excluded) in mechanical CPR do not have decreasing ETCO_2_. No difference in patient outcomes.	
			Thumper was used.			

### Studies comparing quality of CPR

Two manikin studies comparing CPR quality suggested that mechanical CPR performs better during ambulance transport. In the prospective and observational study (N = 144) conducted by Stapleton [18], mechanical CPR was able to provide correct compressions for overall 97% of the runs as compared to 37% of the runs in manual CPR. The 97% rate remained valid for different environmental settings such as vehicle type and road conditions while manual compressions varied upon different settings. Similarly, Sunde [19] noted that compression depth and frequency provided by mechanical CPR devices were better at both cardiac arrest sites and during transport as they were within CPR standards. An increase in weak compressions for manual CPR was observed during ambulance transport. The recommended compression-decompression ratio of 50/50 was only achieved with the device as compared to other techniques where the ratio was significantly lower.

Similar observations were made by Olasveengen [22] where a retrospective and observational study was conducted to evaluate the quality of CPR before and during transport on adult patients with OHCA. In both settings, hands-off ratio for mechanical CPR was shorter than manual CPR and chest compression rates for mechanical CPR were closer to recommended guidelines. Cerebral Performance Categories (CPC) scores were also gathered but no conclusions could be drawn to patient’s outcome due to overall small sample size (N = 75).

However, conflicting results were shown in a prospective and randomized study conducted by Wang [25]. The average chest compression rate and ventilation rate was significantly lower (related to the setting) for mechanical CPR (p < 0.05, p < 0.05) although there was lesser variability in the instantaneous chest compression rate. Due to short transport time of average 4 minutes and small sample size (N = 20), the study indicated no significant difference in average no-chest compression interval.

### Studies comparing clinical outcomes

Many studies did not separate patients who required CPR at scene from those who required it during transport. However, most articles indicated that the patients were loaded with the CPR device onto the ambulance and then transported with CPR ongoing.

A retrospective case–control study (N = 262) conducted by Casner [23] found that for mechanical CPR, higher rates of ROSC was observed in patients overall (39% vs. 29%, p < 0.05) and in asytole (37% vs. 22%, p < 0.05) upon arrival at the Emergency department. There was no significant difference in ROSC for sub-groups with shockable rhythms and PEA. However, these results might be confounded by selective late deployment of the mechanical CPR device in patients not responding to resuscitation.

Ong [12] compared both types of CPR in a phased, non-randomized, observational study (N = 783) of clinical outcomes of patients treated before and after transition from manual CPR to mechanical CPR. The use of mechanical CPR had higher rates of ROSC (34.5% vs. 20.2%, p < 0.05) and survival to hospital admission (20.9% vs. 11.1%, p < 0.05). For survival to hospital discharge, it was better than manual CPR and there was no significant difference in CPC categories (p = 0.36) and Overall Performance Categories (p = 0.40) between both groups.

However, results obtained from Ong [12] were different from a randomized trial (N = 1071) conducted by Hallstrom [13] where the same mechanical CPR device used resulted in worse neurological outcomes and worse survival. Hallstrom’s study found that there was no significant difference between both types of CPR for return of spontaneous circulation 4 hours after the call to 911 was made (29.5% vs. 28.5%, p = 0.74). For mechanical CPR, survival to hospital discharge was poorer (5.8% vs. 9.9%, p = 0.06) and CPC score of 1 or 2 was smaller as compared to manual CPR (3.1% vs. 7.5%, p <0.05).

A similar study conducted by Axelsson [24] showed that there was no significant difference between both types of CPR with regards to ROSC, survival to hospital discharge or to hospital discharge. No difference between CPC score was also observed. Axelsson [20] also evaluated the cardiac output between both types of CPR, measured by pressure of end-tidal carbon dioxide (ETCO_2_). He found that mechanical CPR resulted in higher ETCO_2_ as compared to manual CPR in terms of average values (3.26 vs. 2.69, p < 0.05), initial value (3.38 vs. 2.71, p < 0.05) and minimum value (2.24 vs. 1.69, p < 0.05). No significant differences were observed in maximum value (4.88 vs. 4.48, p = 0.23).

These observations were similarly found in the study initiated by Dickinson [21] who compared whether a mechanical device is better in providing CPR than manual CPR as measured by ETCO_2_ and whether it is capable of improving survival outcomes. In the prospective, odd/even day randomized study conducted (N = 20), he identified that there is sustained or increased ETCO_2_ with the use of mechanical CPR as compared to manual CPR. No difference in patient outcomes was observed between both groups as the study conducted was too small to draw any solid conclusion between two study groups.

## Discussion

Cardiac arrest offers several challenges to the provider when he or she is working to achieve return of spontaneous circulation. Preliminary data suggests that the maintenance of quality CPR declines the moment a patient is moved toward the ambulance or transported to the hospital [26]. Variability in both the depth and the rate of compressions increases dramatically translating to a decline in CPR quality. Beginning from the first patient movement, the provider is often forced to negotiate tight doorways, narrow stairwells, uneven pavement or gravel or dirt just to get the patient to the ambulance. Transporting the patient presents a whole different set of challenges including the rocking of the ambulance, sudden stops, accelerations, decelerations and turns, roadway conditions, not to mention the physical layout of the passenger compartment of the ambulance and how many other providers are present rendering care. Each of these factors contributes in some way to reducing the quality of CPR being delivered. In this paper, a comprehensive review of studies to date relating to the quality of CPR and clinical outcomes when comparing manual to mechanical CPR in OHCA was evaluated.

When considering CPR quality, as defined by current AHA guidelines, results mostly favored mechanical CPR devices. The compression rate and depth was closer to established guidelines utilizing mechanical CPR devices as compared to CPR performed by rescuers. These factors held true through a variety of transport conditions and vehicle types as well. In addition, the compression ratio (number of compressions per minute) was closer to guideline recommendations when mechanical CPR was employed. In the one negative study regarding CPR quality, Wang [25] points out that most of the no-chest-compression intervals as it relates to mechanical CPR were related more to human error and that the compression rate was inadequate because the manufacturer’s default rate was set at 5 compressions to every 1 breath. In addition, although the manual chest compression group in his study had higher instantaneous compression rates, the variability was also higher than that of mechanical CPR group and once the mechanical device was deployed, the instantaneous variability for the mechanical group was lower than the manual CPR group. From this, based upon the studies that were reviewed, mechanical CPR would appear to provide advantages over manual CPR as it relates to instantaneous and continuous CPR fractions throughout all phases of the resuscitation efforts. In addition, mechanical CPR devices appear to provide more consistently appropriate depths of compressions during transport of OHCA patients. However, there is difficulty generalizing the results found here on CPR quality due to small sample sizes, the retrospective nature of one of the studies, and because two of the studies are based upon mannequin evaluations, not real patients.

Clinical outcome measures in OHCA studies often include ROSC, survival to admission, 24-hour survival, and/or survival to discharge. Included in some studies is the CPC score as well as the OPC score. Both of these scores reflect the patient’s ability to function and carry out activities of daily living.

There were six manuscripts that evaluated clinical outcomes in this review. The design of each study was slightly different, making generalizations about clinical outcomes more challenging. In Casner’s [23] and Ong’s [12] manuscripts, there were higher rates of ROSC, with Casner indicating that ROSC was achieved more successfully in asystole. However, the results of Casner’s study have limited validity as late deployment of mechanical CPR devices by responders confounded the evaluation of ROSC because standard CPR was applied before the arrival of device (average 15 minutes). Ong’s [12] larger prospective study demonstrated higher rates of ROSC and survival but no differences in neurological outcomes at discharge. Both of these studies reinforce the importance of consistent high quality and continuous CPR.

Hallstrom’s [13] study was the only randomized controlled trial in this review, but found differing results than Ong’s. A slightly larger study than Ong’s but using the same device, Hallstrom chose to look at ROSC 4 hours after hospital admission. The study was stopped by the safety monitoring committee because of trends toward lower survival rates and poorer outcomes in mechanical CPR patients. Hallstrom’s study design placed his intervention group into several different clusters, which varied the initial resuscitation efforts cluster to cluster. This heterogeneity between clusters makes Hallstrom’s results more difficult to interpret. For example, one of the clusters focused on high quality, early, consistent CPR before the deployment of the Load Distributing Band (LDB) and before any analysis of the rhythm. As a subgroup, this particular cluster trended toward higher rates of ROSC.

Within the past several weeks, and after the preparation of this paper, the Circulation Improving Resuscitation Care (CIRC) trial [27] investigators presented their findings at the 2011 AHA Scientific Sessions in Orlando, Florida in November 2011. This prospective randomized trial of OHCA evaluated mechanical CPR using the LDB compared to “high-quality” manual CPR. The goal of the CIRC trial was to establish equivalence or superiority of one approach over the other. After enrolling over 4000 patients at five sites, and after adjusting for enrolment site, age, witnessed arrest and initial cardiac rhythm, the CIRC investigators found no difference in ROSC 24-hour survival, survival to hospital discharge, and hands-off fraction when comparing mechanical CPR to manual CPR.

Until more evidence is available, it appears that outcomes may not be different between manual and mechanical CPR. We believe that the aim of any pre-hospital service should be to provide the optimum quality of CPR within the constraints and limitations of their operating environment. High quality manual CPR requires a commitment to training, quality review and adequate numbers of staff to attend to cardiac arrests. Mechanical CPR might possibly be helpful as a labor-saving device, but also requires a commitment to training and attention to deployment practices. It might conceivably be helpful from a crew safety viewpoint, reducing likelihood of injuries to crew performing CPR in a moving ambulance. The decision on which to choose would thus depend on the situation of each EMS service.

## Conclusion

In this review, we found insufficient evidence to support or refute the use of mechanical CPR devices in settings of out-of-hospital cardiac arrest and during ambulance transport. While there is some low quality evidence suggesting that mechanical CPR can improve consistency and reduce interruptions in chest compressions, there is no evidence that mechanical CPR devices improve survival, to the contrary they may worsen neurological outcome.

## Abbreviations

AHA, American heart association; CIRC, Circulation improving resuscitation Care; CPC, Cerebral performance categories; CPR, Cardio-pulmonary resuscitation; ETCO2, End-tidal CO2; ILCOR, International liaison committee on resuscitation; LOE, Level of evidence; LDB, Load distributing band; OHCA, Out-of-hospital cardiac arrest; OPC, Overall performance categories; PAROS, Pan asian resuscitation outcomes study; PEA, Pulseless electrical activity; ROSC, Return of spontaneous circulation; VF, Ventricular fibrillation; VT, Ventricular tachycardia.

## Competing interests

For this review paper, it includes information on resuscitation questions developed through the C2010 Consensus on Science and Treatment process, managed by the International Liaison Committee on Resuscitation (http://www.amercian heart.org/ILCOR). The questions were developed by ILCOR Task Forces, using strict conflict of interest guidelines. In general, each question was assigned to two experts to complete a detailed structured review of the literature, and complete a detailed worksheet. Worksheets are discussed at ILCOR meetings to reach consensus and will be published in 2010 as the Consensus on Science and Treatment Recommendations (CoSTR). The conclusions published in the final CoSTR consensus document may differ from the conclusions in this review because the CoSTR consensus will reflect input from other worksheet authors and discussants at the conference, and will take into consideration implementation and feasibility issues as well as new relevant research.

Furthermore, MEH Ong receives grant support from Zoll Medical as principal investigator on several trials associated with load distributing band (LDB) CPR, Autopulse. The names of the trials are as follows:

(1) A Multicenter Randomised Controlled Trial Comparing Shock Success With Synchronized Defibrillation (Compression Upstroke Versus Pre-compression) During Ongoing Mechanical Cardiopulmonary Resuscitation In The Emergency Department

(2) Improving Quality of Cardiopulmonary Resuscitation in the Emergency Department with the Load Distributing Band Device Using Cardiac Arrest Teams and Video Assisted Quality Assurance. A phased non randomised study using historical controls

(3) Comparison Of Load Distributing Band And Standard Cardiopulmonary Resuscitation In Patients Presenting With Cardiac Arrest To Emergency Department

## Authors’ contributions

MEOH and KM conducted the systematic review. ZCZ assisted in the literature search and data extraction. All authors reviewed the data, contributed to and approved the final manuscript.
